# Long-COVID fatigue is not predicted by pre-pandemic plasma IL-6 levels in mild COVID-19

**DOI:** 10.1007/s00011-023-01722-2

**Published:** 2023-03-30

**Authors:** Maxim B. Freidin, Nathan Cheetham, Emma L. Duncan, Claire J. Steves, Katherine J. Doores, Michael H. Malim, Niccolo Rossi, Janet M. Lord, Paul W. Franks, Alessandra Borsini, Isabelle Granville Smith, Mario Falchi, Carmine Pariante, Frances M. K. Williams

**Affiliations:** 1 Department of Twin Research and Genetic Epidemiology, School of Life Course and Population Sciences, King’s College London, London, UK; 2 Department of Biology, School of Biological and Behavioural Sciences, Queen Mary University of London, London, UK; 3 Department of Infectious Diseases, School of Immunology & Microbial Sciences, King’s College London, London, UK; 4 MRC-Versus Arthritis Centre for Musculoskeletal Ageing Research, Institute of Inflammation and Ageing, University of Birmingham, Birmingham, UK; 5 NIHR Birmingham Biomedical Research Centre, University Hospital Birmingham and University of Birmingham, Birmingham, UK; 6 Lund University Diabetes Center, Lund University, Malmö, Sweden; 7 Department of Clinical Sciences, Lund University, Malmö, Sweden; 8 Institute of Psychiatry, Psychology and Neuroscience, King’s College London, London, UK

**Keywords:** Long-COVID, Chronic fatigue, IL-6, Inflammation, BMI

## Abstract

**Objective and design:**

Fatigue is a prominent symptom in the general population and may follow viral infection, including SARS-CoV2 infection which causes COVID-19. Chronic fatigue lasting more than three months is the major symptom of the post-COVID syndrome (known colloquially as long-COVID). The mechanisms underlying long-COVID fatigue are unknown. We hypothesized that the development of long-COVID chronic fatigue is driven by the pro-inflammatory immune status of an individual prior to COVID-19.

**Subjects and methods:**

We analyzed pre-pandemic plasma levels of IL-6, which plays a key role in persistent fatigue, in *N* = 1274 community dwelling adults from TwinsUK. Subsequent COVID-19-positive and -negative participants were categorized based on SARS-CoV-2 antigen and antibody testing. Chronic fatigue was assessed using the Chalder Fatigue Scale.

**Results:**

COVID-19-positive participants exhibited mild disease. Chronic fatigue was a prevalent symptom among this population and significantly higher in positive vs. negative participants (17% vs 11%, respectively; *p* = 0.001). The qualitative nature of chronic fatigue as determined by individual questionnaire responses was similar in positive and negative participants. Pre-pandemic plasma IL-6 levels were positively associated with chronic fatigue in negative, but not positive individuals. Raised BMI was associated with chronic fatigue in positive participants.

**Conclusions:**

Pre-existing increased IL-6 levels may contribute to chronic fatigue symptoms, but there was no increased risk in individuals with mild COVID-19 compared with uninfected individuals. Elevated BMI also increased the risk of chronic fatigue in mild COVID-19, consistent with previous reports.

## Introduction

Fatigue is a common symptom of COVID-19 and is one of its most pronounced acute and post-acute clinical manifestations [1]. Long-lasting manifestations—so-called long-COVID—is a subject of intense interest. Affected individuals report fatigue along with shortness of breath, headache, and loss of sense of taste and smell [2]. Mechanisms underlying the persistence of long-COVID symptoms are yet to be determined. To date, exploration of blood-borne biomarkers following COVID-19 infection has not revealed an association with inflammatory markers or white cell count [3].

The cytokine interleukin (IL)-6 has a recognized role in fatigue development in many clinical settings, including autoimmune inflammatory arthritis (reviewed in [4]) and cancer [5]. It is an established driver of acute responses to COVID-19, and treatment with anti-IL-6 monoclonal antibodies reduces mortality in severe COVID-19 [6, 7]. IL-6 is also considered a key mediator of neuropsychiatric symptoms of long-COVID, including fatigue [8]. Finally, a Mendelian randomization study of depressive symptoms has suggested that IL-6 manifests a causal influence on fatigue, as well as sleep problems and suicidality [9].

We hypothesized that long-COVID fatigue is driven at least in part by the pre-existing immune status of an individual. We have shown previously that chronic fatigue induced by treatment with interferon-alpha (IFN-α) for chronic hepatitis B viral infection is predicted by higher baseline IL-6 and IL-10 levels, as well as an exaggerated elevation of IL-6 and IL-10, in response to treatment [10]. In addition, increasing age is a major risk factor for both illness severity and longevity after SARS-CoV2 infection [2]. Aging has been associated with elevated levels of pro-inflammatory cytokines, such as TNF-α and IL-6, and reduced levels of anti-inflammatory mediator IL-10 [11, 12]. An age-related, chronic, pro-inflammatory milieu may mediate the risk for susceptibility to COVID-19 and long-COVID fatigue. To test this hypothesis, we analyzed levels of pro-inflammatory cytokine IL-6 in plasma collected prior to the SARS-CoV-2 pandemic in a longitudinal cohort sample of UK adults, who were assessed during the pandemic for chronic fatigue symptoms and SARS-CoV-2 infection.

## Materials and methods

### Sample and phenotyping

**Fig. 1:**
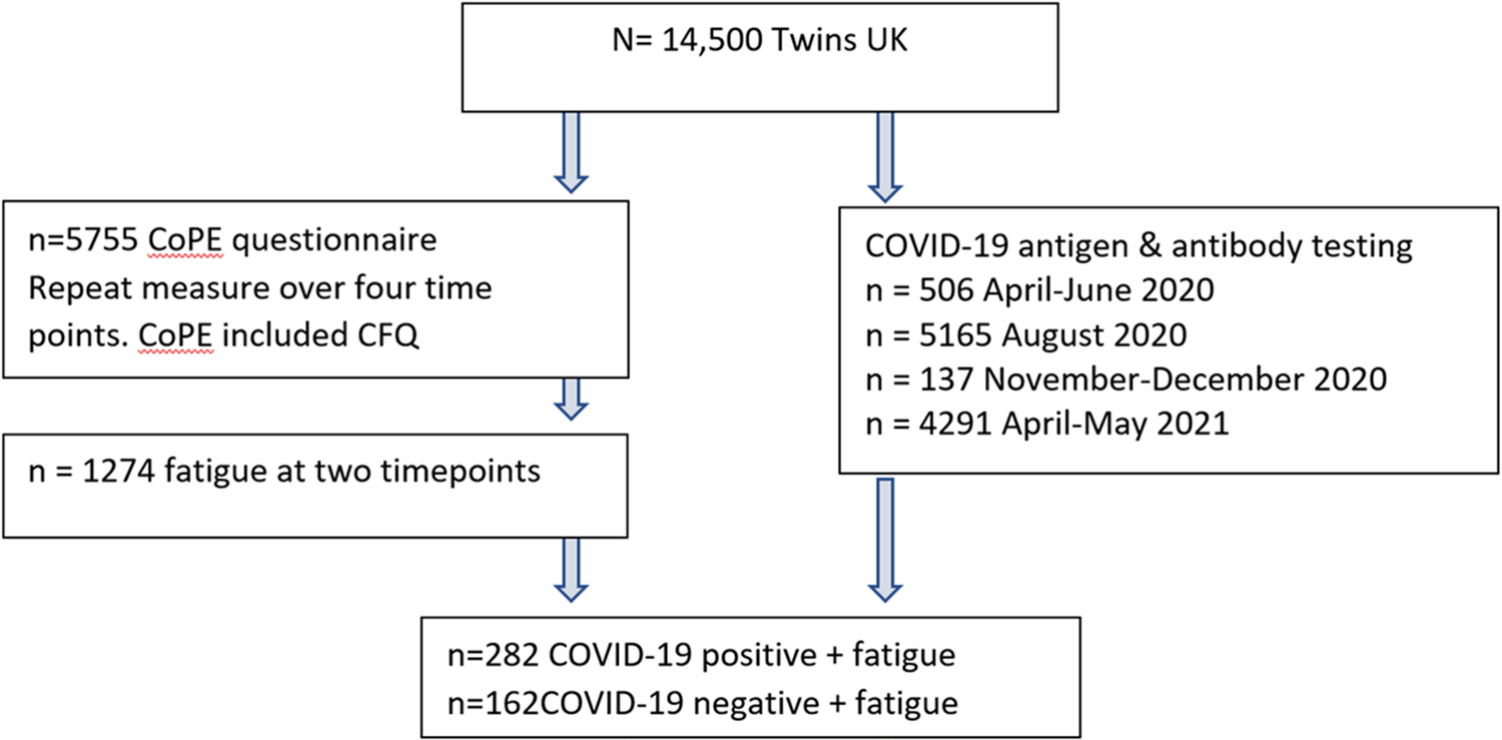
Participant flow diagram. CoPE COVID-19 Personal Experience Questionnaire CFQ Chadler Fatigue Scale

Participants were selected from the UK Twin Registry (TwinsUK), an adult cohort which has been shown to be representative of the general population for lifestyle and health-related traits [13]. Ethics permission was obtained and participants have provided fully informed consent; the Declaration of Helsinki was adhered to. The registry comprises 14,500 same sex mono- and dizygotic twin volunteers from the general UK population recruited from previous twin registers and national media campaigns. The cohort is predominantly female (83%), and mainly of Northern European descent [14]. Samples and data for this project were collected as part of ongoing research initiatives into inflammaging as well as more recent research into COVID-19. Participant selection and grouping is depicted on Fig. 1. Participants (*n* = 5755) were invited to complete a COVID-19 Personal Experience (CoPE) Questionnaire which included questions about COVID-19 infection, related symptoms and fatigue over the previous three months [15]. CoPE was completed in multiple waves during the pandemic: April 2020, August 2020, November 2020, and April 2021. Participants also provided serum for COVID-19 antigen and antibody testing at multiple points during the pandemic, with primary collections of *n* = 506 in April-June 2020, *n* = 5165 in August 2020, *n* = 137 in November–December 2020, and *n* = 4291 in April–May 2021.

We followed guidance on interpretation of antibody test results from the Centres for Disease Control and Prevention (https://www.cdc.gov/coronavirus/2019-ncov/lab/resources/antibody-tests-guidelines.html) to assign natural COVID-19 infection status from the results of swab antigen tests; and enzyme-linked immunosorbent assays (ELISA) anti-Nucleocapsid (anti-N) and anti-Spike (anti-S) Roche antibody tests [16] performed at both King’s College London and 3rd-party laboratories as described previously [17]. Individuals with a positive antigen test at any point over the CoPE questionnaire administration period, who were positive for anti-S antibodies prior to self-reported COVID-19 vaccination date, or positive for anti-N antibodies at any point, were classified as COVID-19-positive cases. Individuals with negative antigen or anti-N antibody results, or negative anti-S antibody results before COVID-19 vaccination, were classified as COVID-19 negative, or controls. Those with negative anti-N antibody and negative antigen results but positive anti-S after COVID-19 vaccination were excluded from the analysis as anti-S antibodies are generated in response to vaccination. Individuals with no laboratory antigen or antibody test results were also excluded from the analysis.

Within the CoPE questionnaire, the Chalder Fatigue Scale (CFQ) [18] was used to classify participants into those experiencing chronic fatigue and those who did not. CFQ comprises 11 questions concerning physical and mental aspects of fatigue. Reliability of CFQ has been shown in clinical and non-clinical settings [18, 19]. CFQ responses were extracted from the CoPE questionnaires administered in August and November 2020. Responses were coded as 0 (“Less than usual”, “No more than usual”) or 1 (“More than usual”, “Much more than usual”) followed by summing the scores for different questions and assigning a diagnosis of fatigue to those with summary score of 4 or more [19]. Volunteers reporting fatigue at both time points were diagnosed as having chronic fatigue because it lasted 3 months or longer. Those who reported fatigue at a single time point were removed from the study.

Pre-pandemic IL-6 levels were ascertained using plasma specimens obtained in 1997–2018 (median 2009). Samples were assayed using Olink Target 96 Inflammation assay (https://www.olink.com/products-services/target/inflammation/). Where multiple plasma specimens were available, we selected the most recent, pre-pandemic specimen. IL-6 levels were expressed as normalized protein expression on the Olink arbitrary unit in log2 scale.

### Statistical analysis

A generalized mixed-effects model with chronic fatigue as a dichotomous categorical response variable and IL-6 levels as a predictor was examined, adjusting for age, body mass index (BMI), and sex as fixed effects. Family structure and zygosity were considered as random factors. IL-6 levels were adjusted for age and BMI followed by transformation of the residuals to a normal distribution using *qqnorm* function in R statistical environment. The model was fitted for COVID-19-positive and COVID-19-negative participants separately. A set of sensitivity analyses was performed. The first investigated sample integrity over time and comprised only plasma samples collected two years before the pandemic; the second was analysis without adjustment of IL-6 levels for age and BMI levels. Finally, we repeated the analysis after excluding individuals with major inflammatory disease (rheumatoid arthritis, systemic lupus erythematosus, ulcerative colitis, and Crohn’s disease; *n* = 11).

## Results

### Prevalence of fatigue

After selecting participants reporting the same fatigue status at both time points, the sample for analysis comprised total *n* = 1274 participants, of whom *n* = 282 were classified COVID-19 positive, and *n* = 162 were classified as having chronic fatigue (Table 1). None of the COVID-19-positive participants had been hospitalized, so their COVID-19 was considered relatively mild. The prevalence of long-term fatigue was 17.4% among COVID-19-positive participants and 11.4% in COVID-19-negative participants (Fisher’s exact *p* = 0.011). COVID-19-positive participants were on average two years younger than COVID-19-negative participants, and there was no difference in BMI observed (Table 1). Two (1.1%) and nine (0.7%) cases of major inflammatory disease were reported by those with and without chronic fatigue, respectively (Fisher’s exact test *p* = 0.641).

**Table 1:** Characteristics of the TwinsUK sample

Group	Sample size	Number with chronic fatigue (%)	Age (SD), years	BMI (SD), kg/m^2^
COVID-19 cases	282	49 (17.4)	65.4 (10.2)	26.5 (5.3)
COVID-19 controls	992	113 (11.4)	67.4 (9.6)	26.2 (4.8)
*p* value		0.011	0.0004	0.356

*p* value provided for comparisons between COVID-19 cases and controls using Fisher’s exact test or Student’s *t* test

*SD* standard deviation

### Structure of fatigue

The qualitative nature of chronic fatigue was similar in COVID-19-positive and -negative participants, with no differences in prevalence of positive answers in the CFQ (Table 2).

**Table 2:** Comparison of Chalder Fatigue Scale responses in COVID-19 cases and controls

Chalder fatigue scale question	COVID-19 cases (*n* = 49)	COVID-19 controls (*n* = 113)	*p* value
Do you have problems with tiredness?	0.68 ± 0.05	0.74 ± 0.03	0.533
Do you need to rest more?	0.70 ± 0.05	0.69 ± 0.03	0.999
Do you feel sleepy or drowsy?	0.60 ± 0.05	0.59 ± 0.03	0.272
Do you have problems starting things?	0.64 ± 0.05	0.70 ± 0.03	0.199
Do you lack energy?	0.82 ± 0.04	0.77 ± 0.03	0.904
Do you have less strength in your muscles?	0.60 ± 0.05	0.56 ± 0.03	0.542
Do you feel weak?	0.48 ± 0.05	0.49 ± 0.03	0.381
Do you have difficulties concentrating?	0.72 ± 0.05	0.65 ± 0.03	0.299
Do you make slips of the tongue when speaking?	0.63 ± 0.05	0.57 ± 0.03	0.902
Do you find it more difficult to find the right word?	0.70 ± 0.05	0.71 ± 0.03	0.896
How is your memory?	0.65 ± 0.05	0.61 ± 0.03	0.345

Prevalence (± standard error) of positive answers among those who was classified as having long-term fatigue is provided; *P* values estimated using Fisher’s exact test

### IL-6 levels

**Fig. 2:**
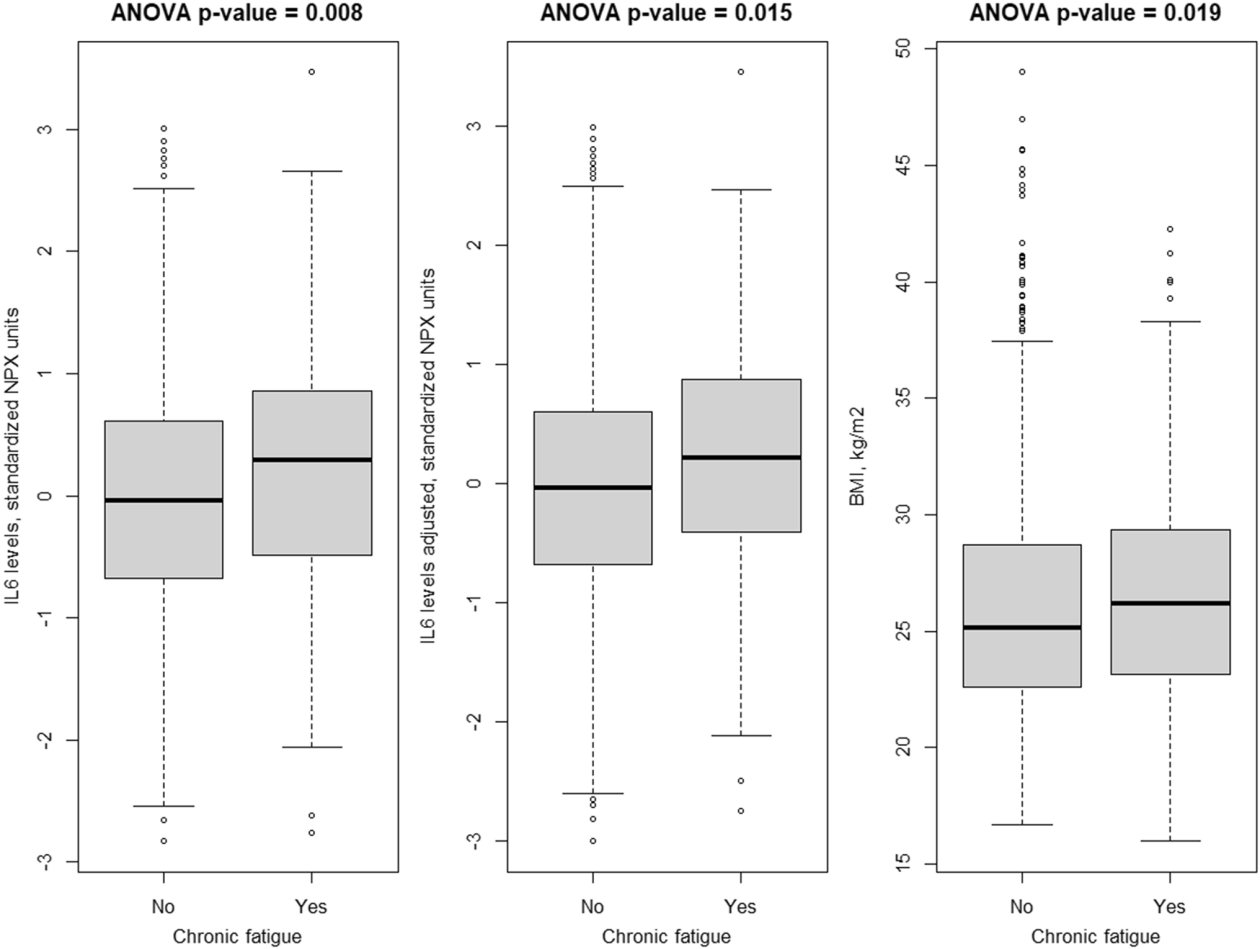
Association between fatigue and pre-pandemic levels of IL-6 and current levels of BMI. First panel shows unadjusted IL-6 values, second panel shows values undusted for age, sex, BMI, and COVID-19 status (via residuals). IL-6 values or the residuals have been transformed to achieve normal distribution

In the total sample, chronic fatigue was associated with elevated pre-pandemic plasma IL-6 levels, which persisted after adjusting cytokine levels for age, sex, BMI, and COVID-19 status; chronic fatigue was as also associated with higher levels of BMI (Fig. 2).

Stratified analysis established that pre-pandemic plasma IL-6 levels were elevated in participants with chronic fatigue in the COVID-19-negative group. The same relationship was not seen in the COVID-19-positive group (Table 3). We also calculated prevalence of fatigue in COVID-19-positive and COVID-19-negative participants stratified by high and low levels of pre-pandemic IL-6. We defined high and low IL-6 levels as values equal to or above 75% percentile of IL-6 distribution and equal to or below 25% percentile, respectively. The prevalence of fatigue was found to be significantly higher in high IL-6 level group compared to low IL-6 level group in COVID-negative participants (17.5% vs 7.6%, *p* = 0.001); however, no such differences were found in COVID-positive group (19.2% vs 19.0%, *p* = 0.999). Sensitivity analysis of participants having plasma collected no earlier than 2017 showed similarity with the main analysis: *β* = 0.653 ± 0.375, *p* = 0.0814; and *β* = − 0.760 ± 0.625, *p* = 0.224 for COVID-19-negative and COVID-19-positive participants, respectively). Sensitivity analysis without adjusting IL-6 levels for age and BMI levels at the time of plasma collection, produced almost identical results to the main analysis: *β* = 0.321 ± 0.115, *p* = 0.005; and *β* = − 0.061 ± 0.180, *p* = 0.774 for COVID-19-negative and COVID-19-positive participants, respectively. Sensitivity analysis with excluding cases of major inflammatory disease, produced almost identical results, too: *β* = 0.307 ± 0.108, *p* = 0.004; and *β* = -0.078 ± 0.166, *p* = 0.639, for COVID-19-negative and COVID-19-positive participants, respectively.

**Table 3:** Risk factors for chronic fatigue in TwinsUK participants by COVID-19 antibody status

Group	Variable	Estimate	Std. error	*z* value	*p* value
COVID-19-positive	(Intercept)	− 1.679	1.356	− 1.238	0.216
	IL-6	− 0.084	0.166	− 0.509	0.611
	Age	− 0.023	0.017	− 1.389	0.165
	Sex	− 1.175	1.051	− 1.118	0.263
	BMI	**0.063**	**0.030**	**2.146**	**0.032**
COVID-19-negative	(Intercept)	− 1.402	0.920	− 1.524	0.127
	IL-6	**0.304**	**0.107**	**2.849**	**0.004**
	Age	− 0.020	0.011	− 1.841	0.066
	Sex	− 0.144	0.510	− 0.282	0.778
	BMI	0.025	0.021	1.170	0.242

Modeling pre-pandemic plasma IL-6 and other factors on the risk of chronic fatigue. IL-6 levels were adjusted for age and BMI followed by transformation to a normal distribution using *qqnorm* function in R. Model was fitted using age and BMI at the time CoPE questionnaire have been administered. Further adjustment was made for family structure and zygosity as random factors

Statistically significant associations are highligted in bold

#### BMI

**Fig. 3:**
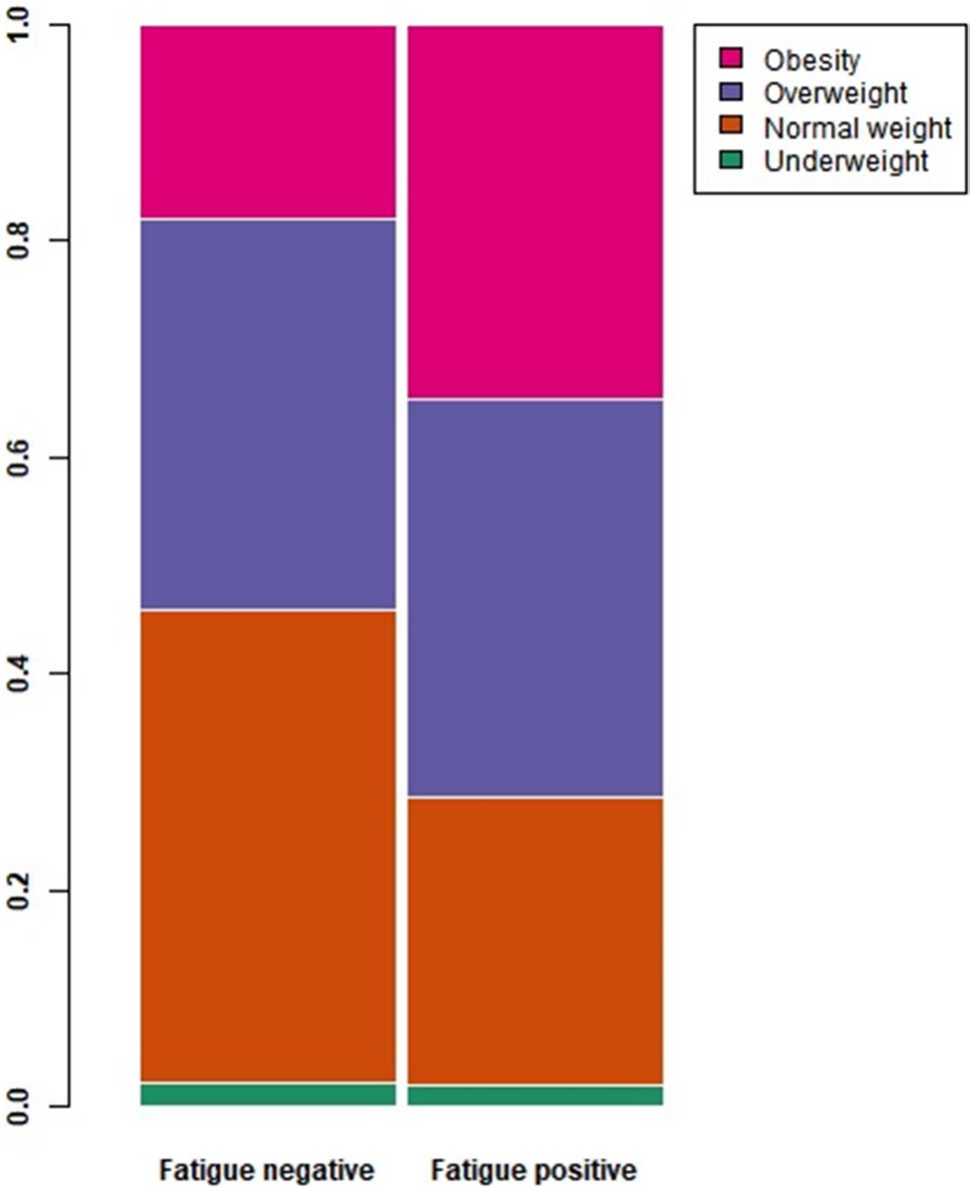
Prevalence of different BMI classes among COVID-19-positive participants with and without chronic fatigue. BMI groups have been defined as the following: underweight (BMI < 18.5; *n* = 6), normal weight (BMI > 18.5 and < 25; *n* = 115), overweight (BMI > 25 and < 30; *n* = 102), and obesity (BMI ≥ 30; *n* = 59). The difference in the prevalence of the BMI groups is statistically significant (*χ*^2^ = 8.3, df = 3, *p* value = 0.040)

Higher BMI was associated with chronic fatigue in COVID-19-positive participants and in the whole sample (Table 3). To explore the relationship between chronic fatigue and BMI in COVID-19-positive participants, we examined groups by BMI (BMI < 18.5; *n* = 6), healthy weight (BMI > 18.5 and < 25; *n* = 115), overweight (BMI > 25 and < 30; *n* = 102), and obese (BMI ≥ 30; *n* = 59). There was a significant difference in prevalence of these groups in COVID-19 positive cases with and without chronic fatigue (*χ*^2^ = 8.3, df = 3, *p* value = 0.040; Fig. 3). This was largely driven by a lower prevalence of healthy weight and higher prevalence of obesity in COVID-19-positive participants with chronic fatigue.

## Discussion

This is among the first studies to examine pre-infective inflammatory cytokine levels and subsequent long-COVID chronic fatigue in participants who experienced mild COVID-19 illness. We found elevated pre-pandemic IL-6 levels increased the risk of developing fatigue in adult volunteers who had never had COVID-19; however, pre-pandemic IL-6 levels did not predict fatigue in our COVID-19-positive group. This finding is in keeping with other post-viral and post-infective fatigue research and studies in a general population [20, 21]. We previously demonstrated that greater pro-inflammatory cytokine elevations in response to IFN-α treatment was associated with developing persistent fatigue [10]. In that study, we also observed a role for pre-treatment higher cytokine levels of IL-6 and lower IL-10 and the development chronic fatigue — which led to the current hypothesis.

While our COVID-19-positive group were not hospitalized and therefore classified with ‘mild’ illness, it is likely that IL-6 and other pro-inflammatory cytokines reach high levels during COVID-19 infection [20], well in excess of levels associated with well-known risk factors such as elevated BMI [21]. Taken together, these and our findings suggest *any* elevation of circulating pro-inflammatory cytokines, be it a protracted, low-grade inflammatory dysregulation or an acute sickness response to infection will increase chronic fatigue risk, with higher cytokine levels associated with higher risk. Baseline immune status or cytokine levels of an apparently healthy individual may only predict chronic fatigue in the absence of pronounced cytokine elevation, such as an acute sickness response to viral or bacterial infection.

Our hypothesis that chronic fatigue in community dwelling adults after SARS-CoV-2 infection was predicated on pre-existing pro-inflammatory immune state) was not supported by our results.

We did however find a statistically significant association between higher BMI and long-COVID fatigue in COVID-19-positive participants (Table 3). This is consistent with a study demonstrating association between obesity and fatigue while controlling for other potential contributors including IL-6 levels [22]. Indeed, almost a third of circulating IL-6 is thought to be produced by adipocytes [23], in keeping with BMI being an important risk factor for chronic fatigue. Of interest, the relationship with BMI and chronic fatigue was not evident in COVID-19-negative participants, an association usually apparent in the population [24].

Allied to this, higher BMI and advanced age are well-established risk factors for severe COVID-19 [22]; and both are associated with greater systemic inflammation [23]. Our findings show that following mild COVID-19 chronic fatigue does not appear to be qualitatively different from other forms of chronic fatigue, at least according to the CFQ, which is a well-validated tool to discriminate between clinical and non-clinical conditions [19]. Whether this is true for more severe disease requiring hospitalization is unclear.

The most pronounced limitation to this study is the focus on a single cytokine and we recognize that other inflammatory mediators such as TNF-α are likely to contribute to chronic fatigue also.

In summary, our work sheds light on the role of IL-6 in general chronic fatigue, but it does not support a specific role for IL-6 levels in the development of chronic fatigue following mild COVID-19.

## Data Availability

The datasets analyzed in the current study are available on request from TwinsUK repository (https://twinsukapps.kcl.ac.uk/data_request).
